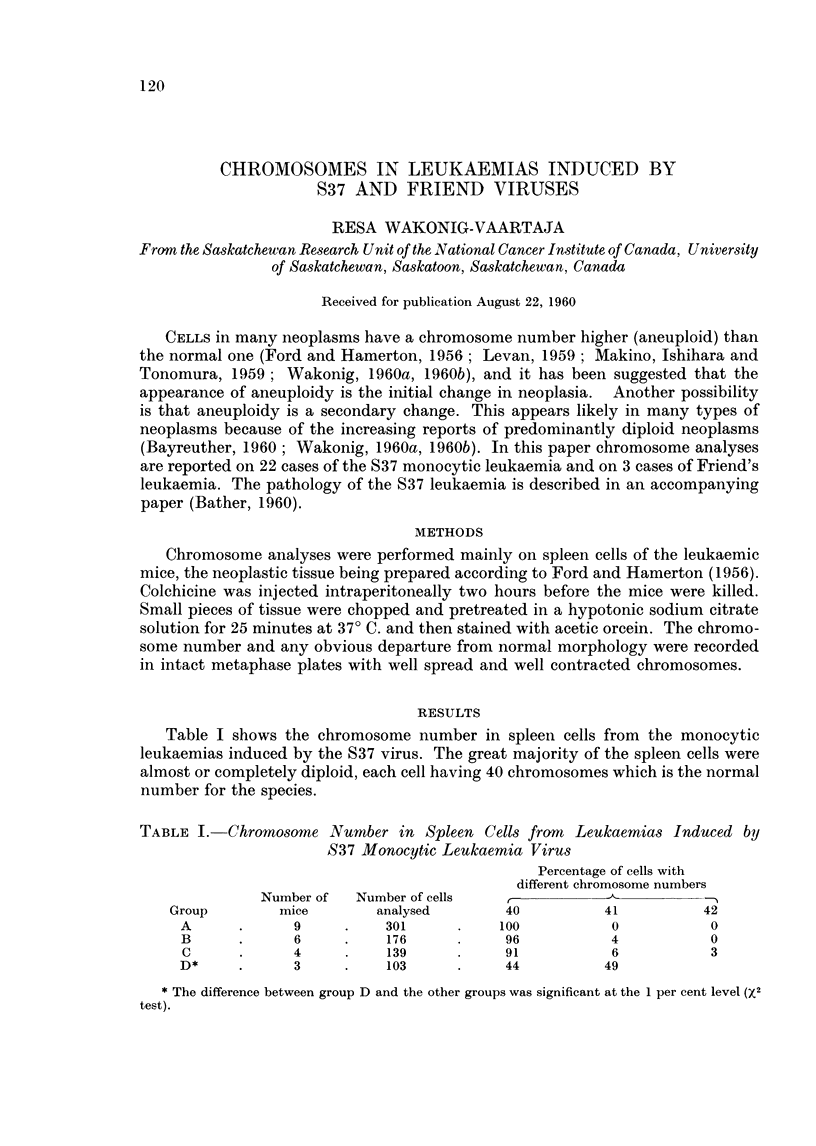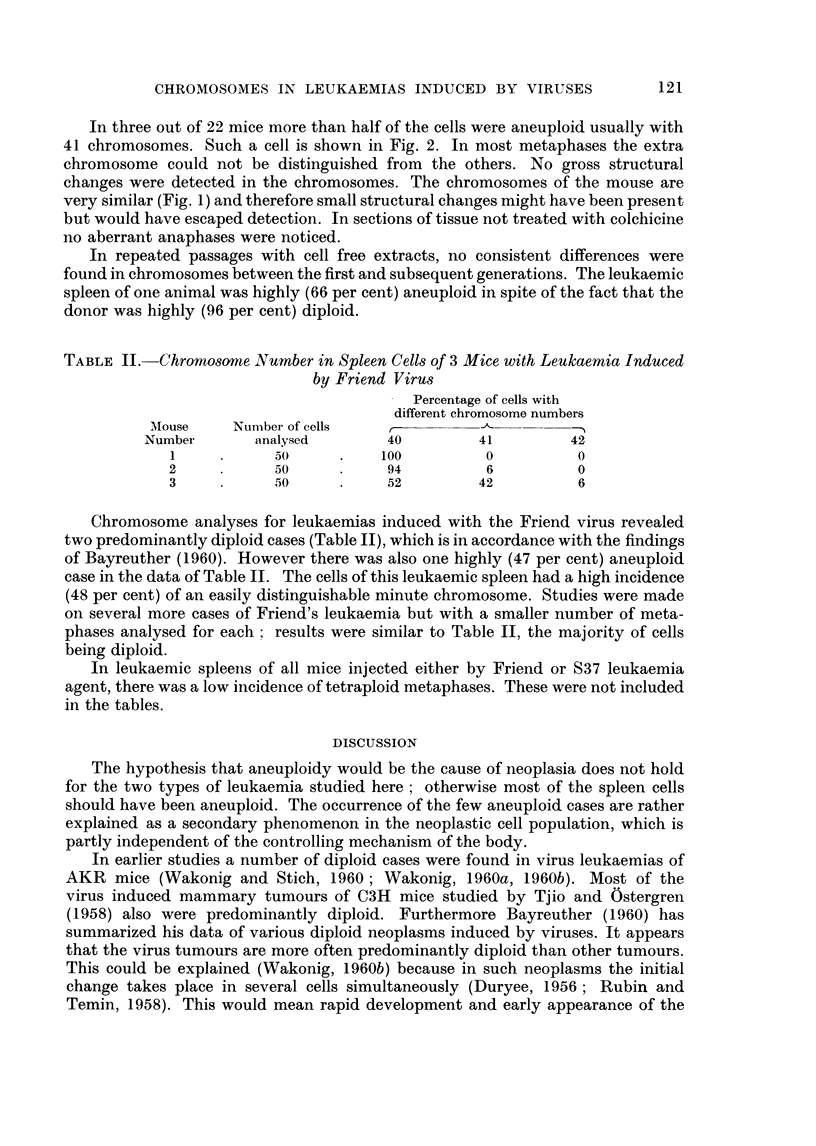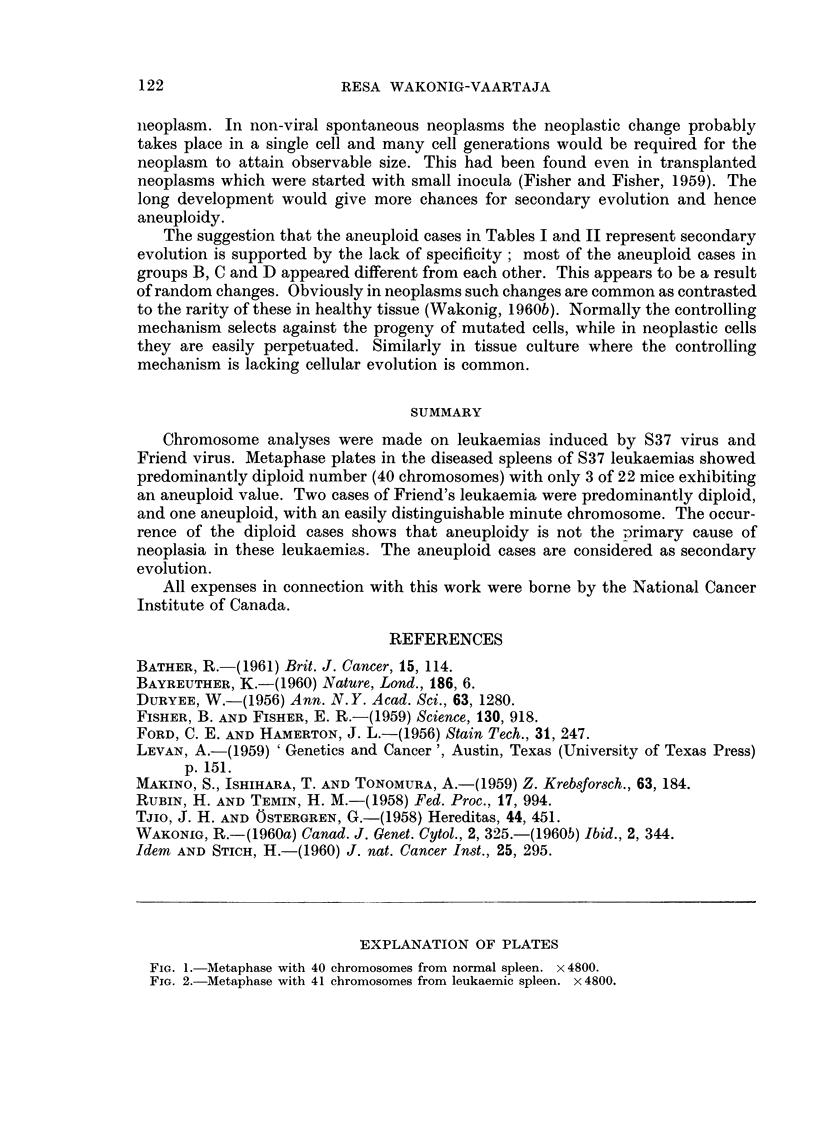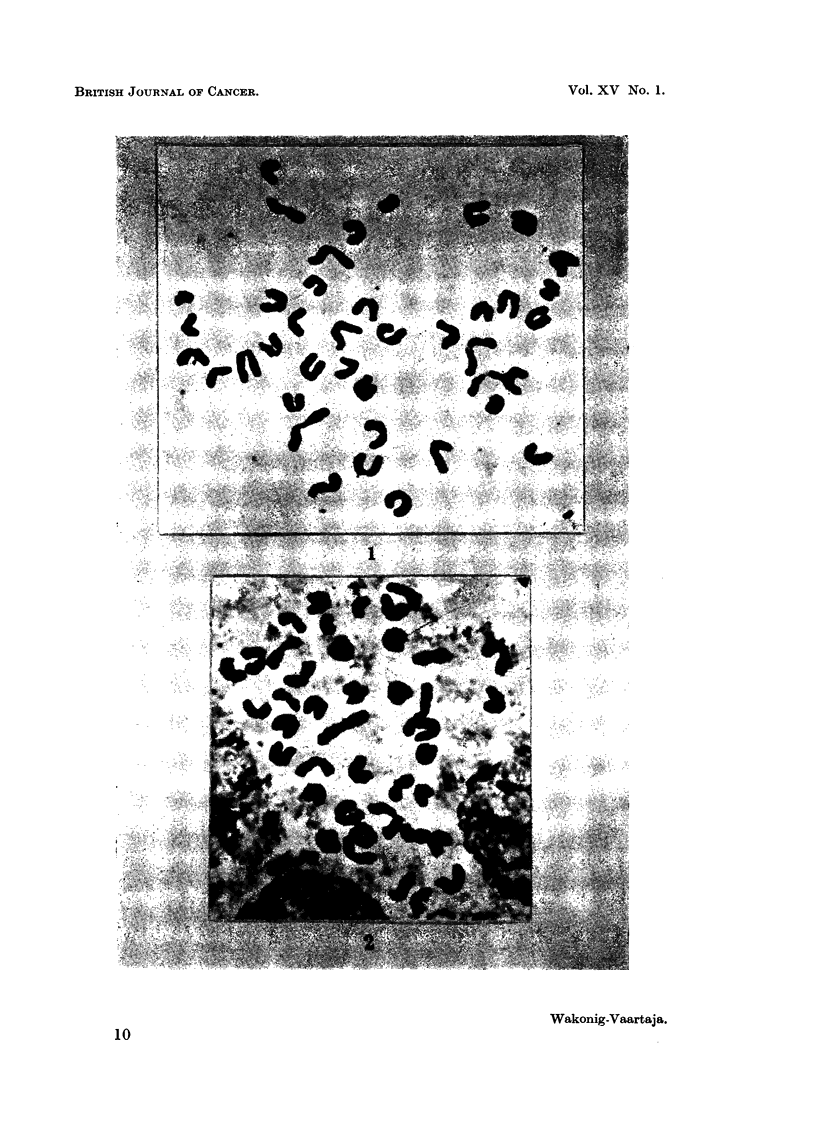# Chromosomes in Leukaemias Induces by S37 and Friend Viruses

**DOI:** 10.1038/bjc.1961.13

**Published:** 1961-03

**Authors:** Resa Wakonig-Vaartaja

## Abstract

**Images:**


### CHROMOSOMES IN LEUKAEMIAS INDUCED BY S37 AND FRIEND VIRUSES

RESA WAKONIG-VAARTAJA

*From the Saskatchewan Research Unit of the National Cancer Institute of Canada, University of Saskatchewan, Saskatoon, Saskatchewan, Canada*

Received for publication August 22, 1960

CELLS in many neoplasms have a chromosome number higher (aneuploid) than the normal one (Ford and Hamerton, 1956 ; Levan, 1959 ; Makino, Ishihara and Tonomura, 1959 ; Wakonig, 1960*a*, 1960*b*), and it has been suggested that the appearance of aneuploidy is the initial change in neoplasia. Another possibility is that aneuploidy is a secondary change. This appears likely in many types of neoplasms because of the increasing reports of predominantly diploid neoplasms (Bayreuther, 1960 ; Wakonig, 1960*a*, 1960*b*). In this paper chromosome analyses are reported on 22 cases of the S37 monocytic leukaemia and on 3 cases of Friend's leukaemia. The pathology of the S37 leukaemia is described in an accompanying paper (Bather, 1960).

### METHODS

Chromosome analyses were performed mainly on spleen cells of the leukaemic mice, the neoplastic tissue being prepared according to Ford and Hamerton (1956). Colchicine was injected intraperitoneally two hours before the mice were killed. Small pieces of tissue were chopped and pretreated in a hypotonic sodium citrate solution for 25 minutes at 37° C. and then stained with acetic orcein. The chromosome number and any obvious departure from normal morphology were recorded in intact metaphase plates with well spread and well contracted chromosomes.

### RESULTS

Table I shows the chromosome number in spleen cells from the monocytic leukaemias induced by the S37 virus. The great majority of the spleen cells were almost or completely diploid, each cell having 40 chromosomes which is the normal number for the species.

TABLE I.—*Chromosome Number in Spleen Cells from Leukaemias Induced by S37 Monocytic Leukaemia Virus*

| Group | Number of mice | Number of cells analysed | Percentage of cells with different chromosome numbers: 40 | 41 | 42 |
|---|---|---|---|---|---|
| A | 9 | 301 | 100 | 0 | 0 |
| B | 6 | 176 | 96 | 4 | 0 |
| C | 4 | 139 | 91 | 6 | 3 |
| D* | 3 | 103 | 44 | 49 | |

* The difference between group D and the other groups was significant at the 1 per cent level ($\chi^2$ test).

In three out of 22 mice more than half of the cells were aneuploid usually with 41 chromosomes. Such a cell is shown in Fig. 2. In most metaphases the extra chromosome could not be distinguished from the others. No gross structural changes were detected in the chromosomes. The chromosomes of the mouse are very similar (Fig. 1) and therefore small structural changes might have been present but would have escaped detection. In sections of tissue not treated with colchicine no aberrant anaphases were noticed.

In repeated passages with cell free extracts, no consistent differences were found in chromosomes between the first and subsequent generations. The leukaemic spleen of one animal was highly (66 per cent) aneuploid in spite of the fact that the donor was highly (96 per cent) diploid.

TABLE II.—*Chromosome Number in Spleen Cells of* 3 *Mice with Leukaemia Induced by Friend Virus*

| Mouse Number | Number of cells analysed | Percentage of cells with different chromosome numbers 40 | 41 | 42 |
|---|---|---|---|---|
| 1 | 50 | 100 | 0 | 0 |
| 2 | 50 | 94 | 6 | 0 |
| 3 | 50 | 52 | 42 | 6 |

Chromosome analyses for leukaemias induced with the Friend virus revealed two predominantly diploid cases (Table II), which is in accordance with the findings of Bayreuther (1960). However there was also one highly (47 per cent) aneuploid case in the data of Table II. The cells of this leukaemic spleen had a high incidence (48 per cent) of an easily distinguishable minute chromosome. Studies were made on several more cases of Friend's leukaemia but with a smaller number of metaphases analysed for each ; results were similar to Table II, the majority of cells being diploid.

In leukaemic spleens of all mice injected either by Friend or S37 leukaemia agent, there was a low incidence of tetraploid metaphases. These were not included in the tables.

### DISCUSSION

The hypothesis that aneuploidy would be the cause of neoplasia does not hold for the two types of leukaemia studied here ; otherwise most of the spleen cells should have been aneuploid. The occurrence of the few aneuploid cases are rather explained as a secondary phenomenon in the neoplastic cell population, which is partly independent of the controlling mechanism of the body.

In earlier studies a number of diploid cases were found in virus leukaemias of AKR mice (Wakonig and Stich, 1960 ; Wakonig, 1960*a*, 1960*b*). Most of the virus induced mammary tumours of C3H mice studied by Tjio and Östergren (1958) also were predominantly diploid. Furthermore Bayreuther (1960) has summarized his data of various diploid neoplasms induced by viruses. It appears that the virus tumours are more often predominantly diploid than other tumours. This could be explained (Wakonig, 1960*b*) because in such neoplasms the initial change takes place in several cells simultaneously (Duryee, 1956 ; Rubin and Temin, 1958). This would mean rapid development and early appearance of the neoplasm. In non-viral spontaneous neoplasms the neoplastic change probably takes place in a single cell and many cell generations would be required for the neoplasm to attain observable size. This had been found even in transplanted neoplasms which were started with small inocula (Fisher and Fisher, 1959). The long development would give more chances for secondary evolution and hence aneuploidy.

The suggestion that the aneuploid cases in Tables I and II represent secondary evolution is supported by the lack of specificity ; most of the aneuploid cases in groups B, C and D appeared different from each other. This appears to be a result of random changes. Obviously in neoplasms such changes are common as contrasted to the rarity of these in healthy tissue (Wakonig, 1960*b*). Normally the controlling mechanism selects against the progeny of mutated cells, while in neoplastic cells they are easily perpetuated. Similarly in tissue culture where the controlling mechanism is lacking cellular evolution is common.

### SUMMARY

Chromosome analyses were made on leukaemias induced by S37 virus and Friend virus. Metaphase plates in the diseased spleens of S37 leukaemias showed predominantly diploid number (40 chromosomes) with only 3 of 22 mice exhibiting an aneuploid value. Two cases of Friend's leukaemia were predominantly diploid, and one aneuploid, with an easily distinguishable minute chromosome. The occurrence of the diploid cases shows that aneuploidy is not the primary cause of neoplasia in these leukaemias. The aneuploid cases are considered as secondary evolution.

All expenses in connection with this work were borne by the National Cancer Institute of Canada.

### REFERENCES

Bather, R.—(1961) *Brit. J. Cancer*, **15**, 114.
Bayreuther, K.—(1960) *Nature, Lond.*, **186**, 6.
Duryee, W.—(1956) *Ann. N.Y. Acad. Sci.*, **63**, 1280.
Fisher, B. and Fisher, E. R.—(1959) *Science*, **130**, 918.
Ford, C. E. and Hamerton, J. L.—(1956) *Stain Tech.*, **31**, 247.
Levan, A.—(1959) ' Genetics and Cancer ', Austin, Texas (University of Texas Press) p. 151.
Makino, S., Ishihara, T. and Tonomura, A.—(1959) *Z. Krebsforsch.*, **63**, 184.
Rubin, H. and Temin, H. M.—(1958) *Fed. Proc.*, **17**, 994.
Tjio, J. H. and Östergren, G.—(1958) Hereditas, **44**, 451.
Wakonig, R.—(1960*a*) *Canad. J. Genet. Cytol.*, **2**, 325.—(1960*b*) *Ibid.*, **2**, 344.
*Idem* and Stich, H.—(1960) *J. nat. Cancer Inst.*, **25**, 295.

---

### EXPLANATION OF PLATES

Fig. 1.—Metaphase with 40 chromosomes from normal spleen. ×4800.
Fig. 2.—Metaphase with 41 chromosomes from leukaemic spleen. ×4800.